# The soluble Decoy Receptor 3 is regulated by a PI3K-dependent mechanism and promotes migration and invasion in renal cell carcinoma

**DOI:** 10.1186/1476-4598-12-120

**Published:** 2013-10-10

**Authors:** Daniel Weissinger, Katrin E Tagscherer, Stephan Macher-Göppinger, Axel Haferkamp, Nina Wagener, Wilfried Roth

**Affiliations:** 1 Molecular Tumor-Pathology, German Cancer Research Center (DKFZ), Heidelberg 69120, Germany; 2 Institute of Pathology, University of Heidelberg, Heidelberg 69120, Germany; 3 Department of Urology, University of Frankfurt am Main, Frankfurt am Main 60590, Germany; 4 Department of Urology, Mannheim Medical Center, University of Heidelberg, Mannheim, Germany; 5 Department of Urology, University of Heidelberg, Heidelberg 69120, Germany

**Keywords:** DcR3, Renal cell carcinoma, AKT, NFAT, Metastasis

## Abstract

**Background:**

Overexpression of Decoy Receptor 3 (DcR3), a soluble member of the tumor necrosis factor receptor superfamily, is a common event in several types of cancer. In renal cell carcinoma (RCC), DcR3 overexpression is associated with lymph node and distant metastasis as well as a poor prognosis. However, the functional role and regulation of DcR3 expression in RCC is so far unknown.

**Methods:**

Modulation of DcR3 expression by siRNA and ectopic gene expression, respectively, was performed in ACHN and 769-P RCC cell lines. Functional effects of a modulated DcR3 expression were analyzed with regard to migration, invasion, adhesion, clonogenicity, and proliferation. Furthermore, quantitative RT-PCR and immunoblot analyses were performed to evaluate the expression of downstream mediators of DcR3. In further experiments, luciferase assays, quantitative RT-PCR and immunoblot analyses were applied to study the regulation of DcR3 expression in RCC. Additionally, an ex vivo tissue slice culture technique combined with immunohistochemistry was used to study the regulation of DcR3 expression in human RCC specimens.

**Results:**

Here, we show that DcR3 promotes adhesion, migration and invasiveness of RCC cells. The DcR3-dependent increase in cellular invasiveness is accompanied with an up-regulation of integrin alpha 4, matrixmetalloproteinase 7 and urokinase plasminogen activator (uPA). Further, we identified a signaling pathway regulating DcR3 expression in RCC. Using in vitro experiments as well as an ex vivo RCC tissue slice culture model, we demonstrate that expression of DcR3 is regulated in a PI3K/AKT-dependent manner involving the transcription factor nuclear factor of activated T-cells (NFAT).

**Conclusions:**

Taken together, our results identify DcR3 as a key driver of tumor cell dissemination and suggest DcR3 as a promising target for rational therapy of RCC.

## Background

Renal cancer accounts for 2.6 percent of all malignant diseases, with RCC (renal cell carcinoma) as the main type of tumor. Around 25% of patients diagnosed with RCC present with advanced disease, including metastasis of the primary tumor. With the onset of metastasis the median survival times for patients range between 10.9 and 29.9 months, depending on the drugs used [1,2]. One reason for the dismal prognosis is the poor response rate to many therapeutic approaches, such as chemotherapy or radiotherapy [3]. The development of (clear cell) renal cell carcinoma is closely linked to the loss of the VHL (von Hippel-Lindau) tumor-suppressor gene, encoding for a protein promoting the degradation of the transcriptional activators HIF1α (hypoxia-inducible factor) and HIF2α. With the loss of VHL, several hypoxia-inducible genes such as VEGF (vascular endothelial growth factor), TGF-α (transforming growth factor α), GLUT-1 (glucose transporter type 1) and carboanhydase 9 are overexpressed and promote tumorigenesis [4-6]. Moreover, activation of the AKT-mTOR (mammalian target of rapamycin) pathway and deregulation of receptor tyrosine kinases contribute to the progression of RCC. These molecular aberrations are targeted by novel therapy strategies such as inhibitors of mTOR or tyrosine kinases [7]. Further, defects in the induction of apoptotic cell death, immune evasion mechanisms and a high metastatic potential are determinants of RCC. In these processes, the members of the TNF (tumor necrosis factor) superfamily play an important role. DcR3 (Decoy Receptor 3) is a soluble member of the TNFR (tumor necrosis factor receptor) superfamily [8]. DcR3 is capable to bind and neutralize CD95 ligand (CD95L, FasL, APO1L), TL1A (TNF-like molecule 1A) and LIGHT. By binding to these ligands DcR3 can inhibit apoptosis, induce angiogenesis and modulate immune cell functions [9-13]. Apart from its decoy function, DcR3 has been shown to induce macrophage differentiation as well as osteoclast formation [14,15]. Clinical data link DcR3 overexpression to different types of cancer, such as pancreatic, lung, hepatocellular and colorectal cancer [8,16-18]. In the tumor entities examined so far, overexpression of DcR3 correlates with higher grading, staging and metastasis [18-21]. In our previous work, we showed that DcR3 expression in RCC is associated with high grade and high stage tumors [19]. Moreover, DcR3 expression correlated with lymph node metastasis and distant metastasis. In addition, DcR3 negatively correlated with disease specific survival and progression free survival and qualified as an independent prognostic factor. In this study, we sought to explore the functional role of DcR3 in RCC. We demonstrate that DcR3 promotes adhesion, migration and invasiveness of RCC cells which is accompanied by an up-regulation of integrin alpha 4, matrixmetalloproteinase 7 and urokinase plasminogen activator (uPA). Further, we show that expression of DcR3 is regulated in a PI3K/AKT-dependent manner. Taken together, our results identify DcR3 as a key driver of tumor cell dissemination and suggest DcR3 as a promising target for rational therapy of RCC.

## Results

### DcR3 promotes migration of RCC cells

**Figure 1:**
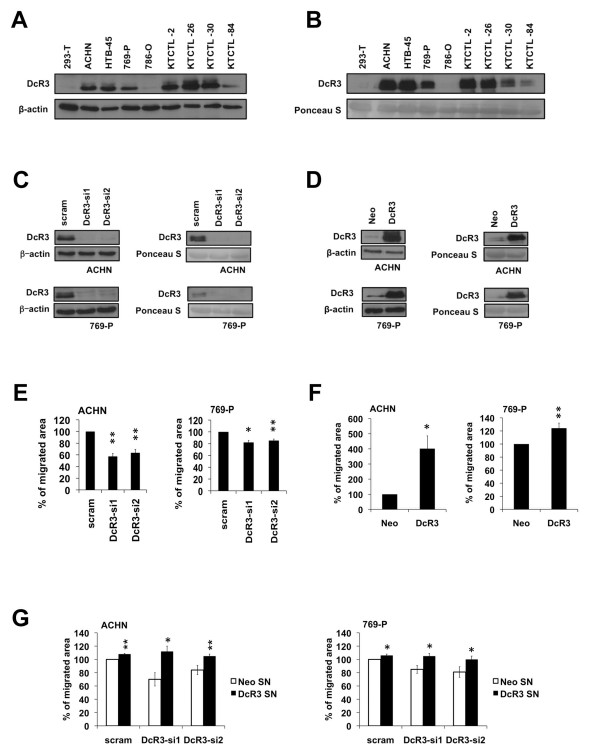
**DcR3 promotes cellular migration in RCC. (A)** Immunoblot analysis of whole-cell lysates showing DcR3 protein levels in different human RCC cell lines and human embryonic kidney 293-T cells. **(B)** Immunoblot analysis of precipitated protein from supernatant showing DcR3 protein secretion in different human RCC cell lines and human embryonic kidney 293-T cells. Ponceau staining is used to demonstrate equal protein loading. **(C**,**D)** Immunoblot analysis of whole-cell lysates (left) and precipitated protein from supernatant (right) of ACHN and 769-P cells transfected with two different DcR3-specific siRNAs or a non-specific siRNA (scram) **(C)** or stably overexpressing DcR3 or an empty control vector (neo) **(D)**. **(E)** Scratch motility assay of ACHN and 769-P cells transfected with two different DcR3-specific siRNAs or a non-specific siRNA (scram). Bars indicate the percentage of cell migration in relation to cells transfected with a non-specific siRNA after 24 h (mean ± SEM; n=3; *p<0.05, **p<0.01; T-test). Representative images are shown in Additional file 1: Figure S1D. **(F)** Scratch motility assay of ACHN and 769-P cells stably overexpressing DcR3 or an empty control vector (neo). Migration was measured over a time course of 12 h (ACHN) or 24 h (769-P). Bars indicate the percentage of cell migration in relation to cells stably transfected with an empty control vector (mean ± SEM; n=3; *p<0.05, **p<0.01, T-test). Representative images are shown in Additional file 1: Figure S1E. **(G)** Scratch motility assay of ACHN and 769-P cells transfected with two different DcR3-specific siRNAs or a non-specific siRNA (scram) and incubated with either DcR3-containing or control supernatant (SN) of stable transfectants. Migration was measured over a time course of 24 h. Bars indicate the percentage of cell migration in relation to cells transfected with a non–specific siRNA and treated with control supernatant (mean ± SEM; n=3; *p<0.05, **p<0.01; T-test).

As our previous work demonstrates a clinical significance of DcR3 overexpression in RCC [19], we were interested in functionally characterizing DcR3 in RCC. To this end, we started to analyze several RCC cell lines for endogenous expression of DcR3 on mRNA and protein level by quantitative RT-PCR and immunoblot analysis. Human embryonic kidney derived 293-T cells were used as a control kidney cell line. Six out of eight RCC cell lines showed a moderate to high expression of DcR3 whereas 293T cells lacked DcR3 expression (Figure 1A; Additional file 1: Figure S1A). As DcR3 is a soluble protein, we additionally investigated its secretion by DcR3 expressing tumor cells. We detected DcR3 in the supernatant of all DcR3 expressing cell lines tested (Figure 1B).

Using these RCC cell lines, we aimed at characterizing the involvement of DcR3 in the regulation of cellular migration, invasion and adhesion. To analyze the effect of DcR3 expression on migratory ability we either downregulated DcR3 using two different siRNAs (Figure 1C) or established transfectants stably overexpressing DcR3 (Figure 1D) and subjected the cells to scratch motility assays. By cytotoxicity analysis we confirmed that modulation of DcR3 expression was functional, as DcR3 overexpression protected cells from CD95L-induced apoptosis, while DcR3 knockdown sensitized cells to CD95L-induced apoptosis (Additional file 1: Figure S1B,C). The siRNA-mediated suppression of DcR3 expression significantly reduced the migratory ability of both cell lines tested (Figure 1E; Additional file 1: Figure S1D), whereas stable overexpression resulted in a strong increase of migration (Figure 1F; Additional file 1: Figure S1E). Consistently, addition of DcR3-containing supernatant rescued the migratory ability of cells with diminished DcR3 expression levels (Figure 1G). To ensure, that our findings are not due to alterations in proliferative capacity, we determined the proliferation rate dependent on DcR3 expression. Downregulation as well as overexpression did not change the proliferative activity nor did it affect clonogenicity (Additional file 2: Figure S2A-D).

### DcR3 increases invasiveness in RCC cells

**Figure 2:**
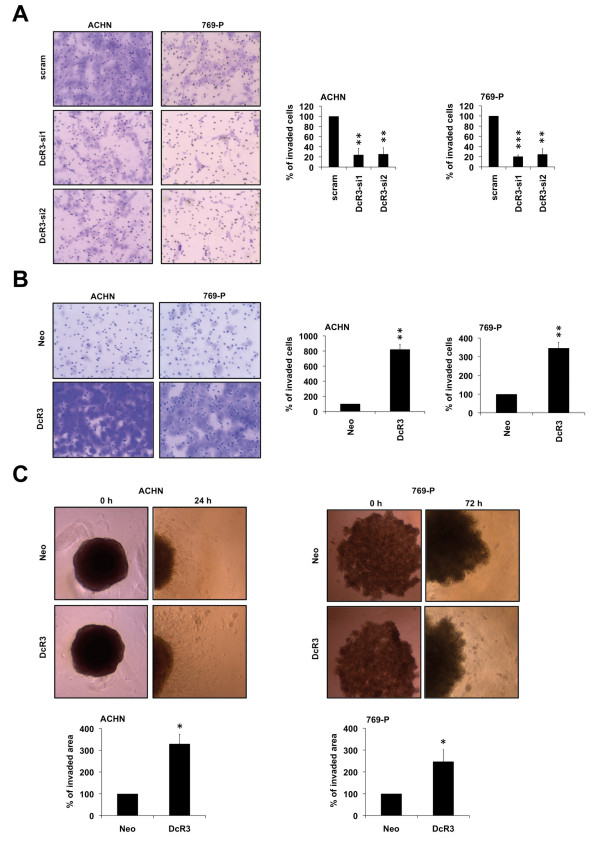
**DcR3 promotes invasiveness in RCC cells. (A**,**B)** Boyden chamber assay of ACHN and 769-P cells transfected with two different DcR3-specific siRNAs or a non-specific siRNA (scram) **(A)** or stably overexpressing DcR3 or an empty vector control (neo) **(B)**. Bars indicate the percentage of cellular invasion in correlation to control cells. Invaded cells were counted in five representative fields (mean ± SEM; n=3, **p<0.01, ***p<0.001; T-test). Representative images are shown (magnification: 200×). **(C)** Spheroid assay of ACHN and 769-P cells stably overexpressing DcR3 or an empty control vector (neo). Bars indicate the percentage of cell invasion in relation to cells stably transfected with an empty control vector (mean ± SEM; n=3; *p<0.05, T-test). Representative images are shown (magnification: 40×).

Next, we tested whether an alteration in DcR3 expression affects the ability of RCC cells to invade the extracellular matrix. While knockdown of DcR3 substantially reduced the invasive capacity (Figure 2A), overexpression strongly enhanced the invasiveness in both cell lines tested (Figure 2B). In addition to the matrigel-coated invasion assay, we studied the invasiveness of RCC cells in a more complex extracellular matrix assay. Cells were grown to form spheroids, which were then implanted into a collagen type I gel-matrix. In line with the matrigel invasion results, overexpression of DcR3 significantly enhanced the invasive phenotype of both cell lines tested (Figure 2C).

### Regulation of cellular adhesion to fibronectin by DcR3

**Figure 3:**
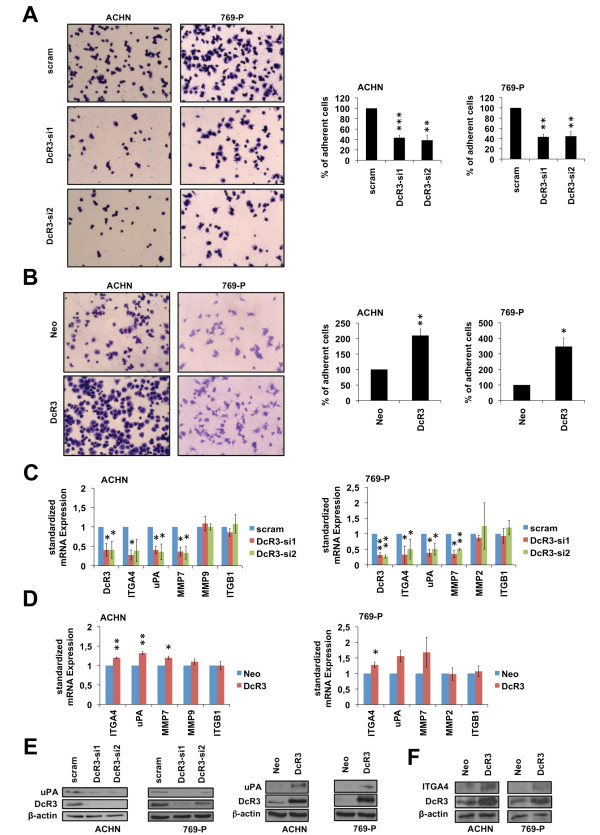
**DcR3 modulates cell adhesion to ECM component fibronectin. (A**,**B)** Adhesion assay of ACHN and 769-P cells transfected with two different DcR3-specific siRNAs or a non-specific siRNA (scram) **(A)** or stably overexpressing DcR3 or an empty vector control (neo) **(B)**. Bars indicate the percentage of cell adhesion compared to control cells. Adherent cells were counted in five representative fields (mean ± SEM; n=3, *p<0.05, **p<0.01; ***p<0.001; T-test). Representative images are shown (magnification: 100×). **(C**,**D)** Quantitative real-time-PCR assaying relative expression levels of DcR3 and invasion-relevant genes in ACHN and 769-P cells transfected with two different DcR3-specific siRNAs or a non-specific siRNA (scram) **(C)** or stably overexpressing DcR3 or an empty vector control (neo) **(D)**. Expression data were normalized to internal 18S rRNA expression. Bars indicate fold changes of RNA level in relation to control cells (mean ± SEM; n=3; *p<0.05; **p<0.01; T-test). **(E)** Immunoblot analysis of whole-cell lysates showing uPA protein level in ACHN and 769-P cells transfected with two different DcR3-specific siRNAs, a non-specific siRNA (scram) or stably overexpressing DcR3 or empty vector control (neo). **(F)** Immunoblot analysis of whole-cell lysates showing ITGA4 protein level in ACHN and 769-P cells stably overexpressing DcR3 or an empty vector control (neo).

As both migration and invasion are dynamic processes involving attachment and detachment to extracellular matrix proteins, we wondered whether the alteration of DcR3 expression might have effects on cellular adherence. To this end, we analyzed the ability of cells with modulated DcR3 expression to attach to cover glasses coated with fibronectin, which is present in RCC [22] and metastatic niches [23]. Interestingly, DcR3 knockdown decreased the ability to adhere to fibronectin (Figure 3A), while overexpression augmented adherence (Figure 3B). Based on these results, we wondered whether DcR3 induces the expression of genes commonly associated with migration, invasion or adhesion. Interestingly we found a DcR3 dependent alteration of expression levels for ITGA4 (integrin alpha 4), MMP7 (matrixmetalloproteinase 7) and uPA (urokinase plasminogen activator) whereas expression levels of ITGB1 (integrin beta 1), MMP2 and MMP9 were unchanged (Figure 3C-F).

### PI3K/AKT signaling regulates DcR3 expression in RCC

**Figure 4:**
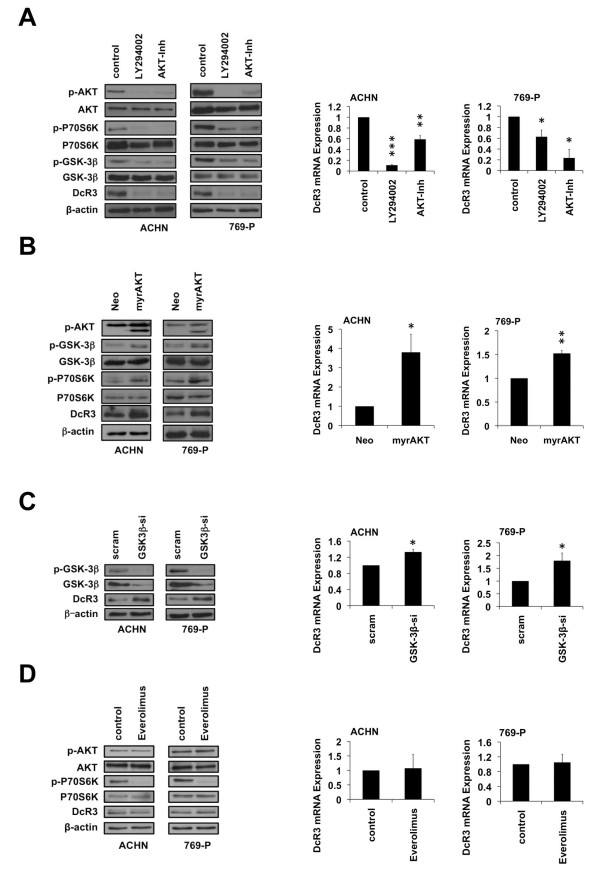
**DcR3 expression in RCC is PI3K/AKT-dependent. (A**-**D)** Immunoblot analysis of whole-cell lysates and quantitative real-time-PCR assaying relative DcR3 mRNA expression of ACHN and 769-P cells 24 h after treatment with LY294002 (50 μM) or AKT-inhibitor IV (10 μM) **(A)**; 48 h post transfection with constitutively active AKT (myrAKT) or an empty vector control (neo) **(B)**; 48 h post transfection with GSK-3β-specific siRNAs or a non-specific siRNA (scram) **(C)**; 24 h after treatment with Everolimus (1 μM) **(D)**. Expression data were normalized to internal 18S rRNA expression (mean ± SEM; n=3; *p<0.05, **p<0.01, ***p<0.001; T-test).

Both the expression data derived from human RCC samples [19] as well as the functional results obtained in the cell culture model indicate a key role of DcR3 in the process of invasion and metastasis. However, the mechanisms responsible for overexpression of DcR3 in RCC are not known. Since the PI3K/AKT pathway is deregulated in RCC [24], we investigated its involvement in the regulation of DcR3 expression. Treatment of RCC cell lines with both the PI3K-inhibitor LY294002 and the AKT-inhibitor IV (CAS-number: 681281-88-9) resulted in a strongly reduced DcR3 expression on both protein and mRNA level, indicating a regulation of DcR3 on the transcriptional level (Figure 4A). Correspondingly, overexpression of the constitutively active form of AKT led to an increased DcR3 expression (Figure 4B). The successful modulation of the PI3K/AKT pathway was further confirmed by analyzing the phosphorylation of AKT, its direct downstream target GSK-3β, the mTOR target P70S6K (Figure 4A,B) and by measuring the activity of the FOXO transcription factors (Additional file 3: Figure S3A,3B). We further evaluated the role of GSK-3β and mTOR in the PI3K/AKT-dependent DcR3 regulation. Knockdown of GSK-3β, whose activity is negatively regulated by AKT, resulted in a moderate increase of DcR3 expression (Figure 4C). In contrast, the inhibition of mTOR using Everolimus had no impact on DcR3 expression (Figure 4D).

### NFATc1 mediates PI3K/AKT-dependent DcR3 expression

**Figure 5:**
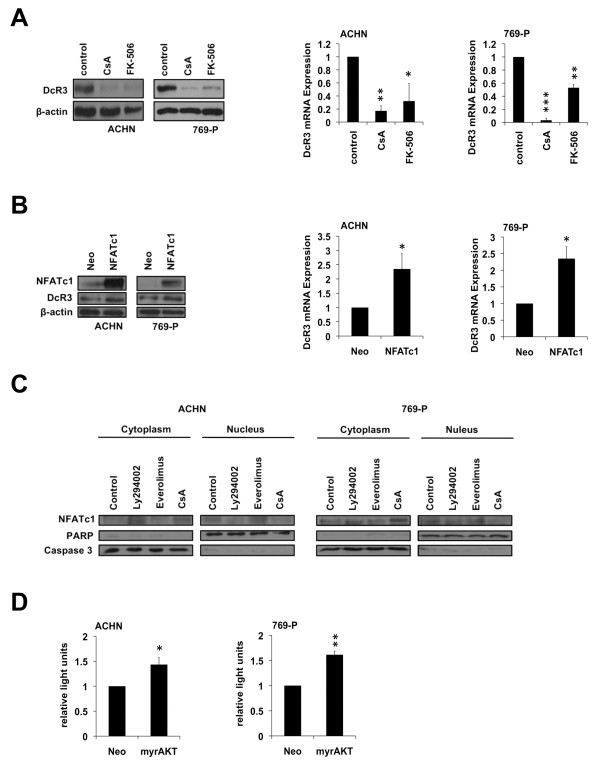
**NFATc1 regulates DcR3 expression at a transcriptional level. (A**,**B)** Immunoblot analysis of whole-cell lysates and quantitative real-time-PCR assaying relative DcR3 mRNA expression of ACHN and 769-P cells 24 h after treatment with cyclosporin A (CsA, 25 μM) or Tacrolimus (FK-506, 50 μM) **(A)**; 48 h post transfection with NFATc1 or an empty vector control (neo) **(B)**. Expression data were normalized to internal 18S rRNA expression (mean ± SEM; n=3; *p<0.05, **p<0.01, ***p<0.001; T-test). **(C)** Immunoblot analysis of cytoplasmic and nuclear fractions of ACHN and 769-P cells after treatment with LY294002 (50 μM), Everolimus (1 μM), or Cyclosporine A (25 μM). **(D)** Relative NFATc1–luciferase reporter activity of ACHN and 769-P cells 24 h post transfection with myrAkt or an empty vector control (neo) (mean ± SEM; n=3; *p<0.05, **p<0.01; T-test).

GSK-3β and the family of FOXO transcription factors are both known to negatively regulate the transcription factor NFAT (nuclear factor of activated T-cells) [25]. Therefore, we investigated its role in the transcriptional regulation of DcR3. We treated the cells with Cyclosporine A or FK-506 (Tacrolimus) which are both immunosuppressants that inactivate calcineurin, the major activator of NFAT. Inhibition of calcineurin dramatically decreased the expression of DcR3 (Figure 5A), indicating a functional relevance of NFAT in DcR3 regulation. Accordingly, NFAT overexpression resulted in an increase in DcR3 expression level (Figure 5B). To demonstrate that modulation of the PI3K/AKT pathway affects NFAT expression, we performed nuclear and cytoplasmic fractionation and detected a shift of NFAT localization to the cytoplasm upon PI3K inhibition. A similar shift was detectable after Cyclosporine A treatment which served as a positive control. In contrast, treatment with Everolimus had no impact on NFAT localization, confirming an mTOR independent regulation (Figure 5C). In addition, the activity of NFAT was enhanced upon overexpression of a constitutively active form of AKT (Figure 5D).

### PI3K/AKT signaling regulates DcR3 expression in ex vivo cultured RCC tissue

**Figure 6:**
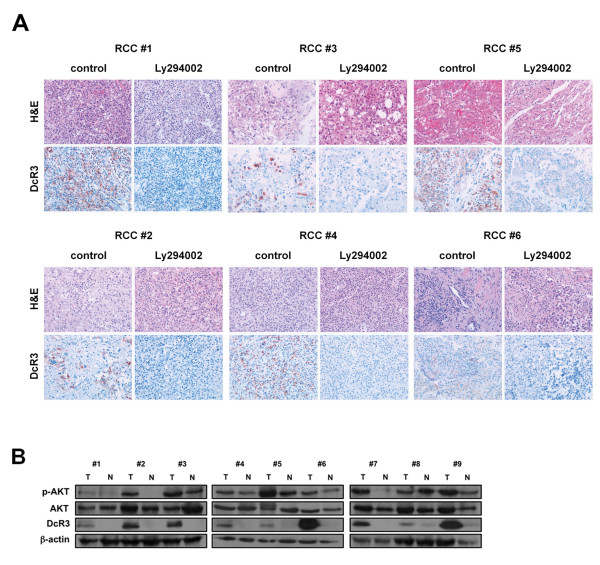
**DcR3 expression in human RCC specimens is PI3K/AKT-dependent. (A)** Immunohistochemical analysis of DcR3 expression in *ex vivo* tissue samples 48 h after treatment with LY294002 (100 μM). Representative images are shown (magnification: 100×). **(B)** Immunoblot analysis of DcR3 and phospho-AKT protein levels in nine different pairs of RCC and respective normal renal tissue.

To confirm the importance of PI3K signaling for DcR3 expression in human RCC, we incubated freshly resected human RCC tissue slices with the PI3K-inhibitor LY294002. The inhibition of PI3K signaling significantly diminished DcR3 expression in all six examined cases, as assessed by immunohistochemistry (Figure 6A). These results were confirmed by immunoblot analyses of lysates generated in parallel (Additional file 3: Figure S3C). Furthermore, treatment of RCC tissue slices with LY294002 resulted in a reduced proliferation in four out of five cases as assessed by Ki-67 staining. At the same time, apoptosis (assessed by immunohistochemical detection of cleaved caspase 3) was not induced to a significant extent by LY294002 (data not shown).To further examine a possible association of AKT activation levels and DcR3 expression, we subjected nine pairs of freshly obtained human RCC tissue and adjacent normal renal tissue to immunoblot analysis. Although a clear quantitative association of AKT phosphorylation and DcR3 expression levels was not evident, the majority of DcR3 positive tumor samples also showed elevated levels of active AKT compared to their corresponding normal tissue samples (Figure 6B).

## Discussion

In our previous work we found a significant association of DcR3 expression levels and both lymph node and distant metastasis in a large collection of 560 human RCC samples [19]. Further, DcR3 expression was identified as a robust independent negative prognostic marker in patients with RCCs. In the present study we sought to elucidate the functional relevance of DcR3 for cellular migration, invasiveness and metastasis. Moreover, we investigated the mechanisms of how DcR3 expression is regulated in RCC.

Our results indicate that DcR3 is an important driver of adhesion, migration and invasiveness in RCC. Since these functional characteristics are hallmarks of the metastatic process the findings are in accordance with the clinical correlation of DcR3 expression and metastasis. Similar results obtained by studies of other types of cancer, such as breast and nasopharyngeal cancer, confirm the promoting effect on metastasis and invasiveness of DcR3 [26,27]. In addition to our functional observations, we found that DcR3 regulates the expression of proteins involved in migration and invasiveness. Modulation of DcR3 expression resulted specifically in transcriptional regulation of MMP-7, uPA and ITGA4, whereas the expression of other members of the matrixmetalloproteinase and integrin families was not altered. In a recent study of ovarian cancer, DcR3 overexpression was shown to regulate a whole network of proteins. ITGA4, uPA and members of the MMP-family were positively regulated by DcR3 [28]. Additionally, DcR3 was shown to upregulate ITGA4 in macrophages [29]. These data provide additional evidence that DcR3 is involved in the induction of metastasis–associated genes. Interestingly, MMP7, uPA and ITGA4 have been shown to correlate with metastatic potential in RCC. ITGA4 is exclusively expressed in RCC in comparison to normal kidney tissue [30] and is associated with metastatic spread of RCC and other solid tumor entities by interacting with its ligands VCAM-1 (vascular cell adhesion protein 1) and fibronectin [31,32]. VCAM-1 and ICAM-1 (intercellular adhesion molecule 1) are other proteins that were shown to be upregulated upon DcR3 exposure on endothelial cells [33]. Since the interaction of ITGA4 with VCAM-1 is essential for the leukocyte adhesion cascade involving rolling, adhesion and transmigration through endothelial cells, DcR3 might enable cancer cells to mimicry this process in order to form distant metastasis. Such mimicry effect has already been shown upon TNF-α (tumor necrosis factor α) stimulation in oral squamous cell carcinoma [34]. In addition, MMP-7 and uPA expression correlate with metastasis and poor survival rates in RCC [35,36]. The precise mechanism of DcR3 signaling remains unknown but could involve binding to the heparan sulfate proteoglycans syndecan-2 and CD44v3, both exerting downstream effects on Src (Rous sarcoma oncogene cellular homolog), Ras and consequently STAT3 (signal transducer and activator of transcription 3) signaling [37-40]. In our experiments we could confirm a role of STAT3 in DcR3 signaling (data not shown), whereas Src amongst other pathways such as PKC (protein kinase C)-, PI3K- and FAK (fokal adhesion kinase)-dependent signaling is influenced by DcR3 in immune cell response [41]. Since both MMP-7 and ITGA4 are transcriptionally regulated by STAT3, Src/STAT3 signaling might explain the transcriptional regulation of MMP-7 and ITGA4 in the context of DcR3 [42,43].

The mechanisms of regulation of DcR3 expression in RCC have not yet been investigated. Our study demonstrates that DcR3 expression is regulated by a PI3K/AKT-dependent mechanism. In human pancreatic adenocarcinoma, DcR3 expression has been linked to PI3K/AKT signaling in cooperation with NFκB (nuclear factor κB), however, without further investigation of possible downstream mediators [44]. Another study linked Epstein-Barr virus transcription activator Rta to PI3K/AKT and NFκB signaling and increased DcR3 expression [45]. As AKT influences a whole network of proteins and interacts with different other pathways we evaluated the role of two major AKT downstream targets. Thereby we could exclude mTOR as a major regulator of DcR3 expression. In contrast, GSK-3β turned out to be involved in the regulation of DcR3 expression. GSK-3β is capable of inactivating the transcription factor NFAT by phosphorylation, leading to a translocation of NFAT into the cytoplasm, which renders it unable to induce transcription of its downstream targets [25]. FOXO can increase the expression of atrogin-1, which is able to ubiquitinate calcineurin, consequently leading to a decrease in NFAT activation [46]. Via further experiments, we could describe NFAT as the main driver of DcR3 expression. Recently, possible cross-talks between NFAT and NFκB were described in bronchial epithelial cells [47] and in cardiomyocytes [48]. In line with these observations, we observed a decreased expression of DcR3 upon p65/RelA knockdown (data not shown). Since the PI3K/AKT pathway is able to positively regulate NFκB signaling [49], the PI3K/AKT/NFAT-mediated regulation of DcR3 might additionally be enhanced by the PI3K/AKT/NFκB axis. As the TNF superfamily shows structural similarities, one might assume similar mechanisms regarding regulation of expression. Interestingly, the soluble TNF superfamily members TRAIL (TNF-related apoptosis inducing ligand), CD95L, RANK-L (receptor activator of NFκB ligand) and TNF-α are upregulated by NFAT and SP-1 [50-54]. In contrast, the role of NFAT in the regulation of death receptors has not been examined in detail so far. SP-1 was reported to upregulate the expression of TRAIL-R2 (DR5; death receptor 5), while analysis of the other TNFR members is still missing [55]. In line with these observations we could also detect a decrease of DcR3 expression upon SP-1 inhibition or SP-1 knockdown (data not shown). Thus, similar mechanisms regulate the expression of different members of the TNF and TNFR superfamilies.

As DcR3 emerges as a multimodal molecule capable of promoting tumor progression by neutralizing apoptosis-inducing ligands, modulating the immune system in a pro-tumorigenic way as well as promoting metastasis of cancer cells, DcR3 might be a promising therapeutic target. The fact, that DcR3 is a secreted protein implies that DcR3 is capable of acting on its different target cells in a paracrine as well as a systemic manner, rendering it a powerful tool of the tumor to modulate the host system to form metastasis. On the other side, DcR3 is easily detectable in the blood serum of patients, opening up the possibility to use DcR3 as a biomarker for risk of metastasis and aggressive disease. Moreover, DcR3 expression could qualify as an indicator for the response and efficacy of a PI3K/AKT-targeting therapy. If the correlation between DcR3 expression and PI3K/AKT signaling activities proves to be stable in patients, the evaluation of DcR3 levels in urine or blood samples of patients could allow a selection of patients for treatment with PI3K/AKT-targeting therapeutics such as NVP-BEZ235 or SF1126 which are already in clinical trials for RCC and other solid tumor entities (NCT01453595; NCT00907205). SF1126 is a RGDS-conjugated LY294002 prodrug which is converted to the active compound LY294002 under physiologic conditions [56]. SF1126 is characterized by an increased solubility, by a prolonged half-life and by an improved delivery to the tumor [56]. It was recently evaluated in a phase I clinical trial in advanced solid tumors and B cell malignancies and has been proven to be well tolerated [57]. However, similarly to Ly294002 [58], SF1126 is likely to target signaling pathways other than PI3K/AKT, such as GSK3, mTOR and PI4K. So far the efficacy of a PI3K/AKT-targeting therapy in patients with RCC is not clear. DcR3 could serve as an easily measurable indicator for response, as DcR3 protein level should decrease upon PI3K/AKT inhibition. PI3K/AKT activation is a common event in cancer progression, either by loss of PTEN (phosphatase and tensin homolog) or activation of receptor tyrosine kinases by stimuli such as TGF-α or VEGF [59] the latter being highly upregulated in clear cell renal cancer. Consequently, PI3K/AKT-signaling is highly active in RCC and correlates with tumor burden and metastasis [60]. Thus, our findings suggest a new role of DcR3 in the context of rationally treating RCC patients. Our study warrants the further investigation of DcR3 in clinical settings.

## Conclusions

This is the first study on the regulation and function of DcR3 in RCC. Our experiments identify DcR3 as a driver of adhesion, migration and invasiveness in RCC cells. These results confirm previous data demonstrating that DcR3 expression can be used as a biomarker for poor prognosis and risk of metastasis. Furthermore, we identified that DcR3 is regulated via a signaling axis involving PI3K/AKT and NFATc1. This newly discovered signaling pathway could be therapeutically exploited, since the subgroup of patients with DcR3 positive RCC might benefit from the inhibition of the drugable targets PI3K/AKT and NFATc1.

## Methods

### Reagents

Everolimus (#07741) was obtained from Sigma Aldrich (St. Gallen, Switzerland), LY294002 (#70920) was purchased from Cayman Chemicals Company (Ann Arbor, MI, USA) and AKT-inhibitor IV (#124011) was purchased from Calbiochem (Darmstadt, Germany). Cyclosporine A (#BML-A195) and CD95L (#ALX-522-001) were bought from Enzo life science (Loerrach, Germany). FK-506/Tacrolimus (#10007965) was purchased from Biozol (Eching, Germany).

### Cell culture

Human RCC cell lines 786-O, 769-P, ACHN, HTB-45 and 293-T were purchased from ATCC (Rockville, MD, USA). At ATCC the cell lines were authenticated by short tandem repeat profiling. KTCTL-2, KTCTL-26, KTCTL-30 and KTCTL-84 were obtained from the tumor cell bank of the German cancer research center (DKFZ; Heidelberg, Germany). All cell lines were maintained in RPMI1640 medium (Life Technologies, Gaithersburg, MD, USA) supplemented with 10% fetal calf serum, 1 mM glutamine, 25 mM glucose and 1% penicillin/streptomycin (Life Technologies) and cultured at 37°C in a 5% CO_2_ atmosphere. For experiments, cells were cultured for no more than 10 passages. In addition, cell lines were regularly tested for contamination by multiplex PCR performed in the Genomics and Proteomics Core Facility [61] (DKFZ, Heidelberg, Germany). Trypan blue exclusion assay was used for cell viability evaluation.

### Transfections

Knockdown of endogenous protein was achieved by transiently transfecting cell lines with short interfering RNA (siRNA) oligonucleotides at a concentration of 25 nM using Lipofectamine 2000 (Invitrogen, Carlsbad, CA, USA). All siRNAs were obtained from Dharmacon (Lafayette, CO, USA): DcR3_si1 (#D-008102-02), DcR3_si2 (#D-008102-04), GSK-3β_si (#J-00310-12). A non-specific siRNA served as a control (#D-001810-10). For transient or stable overexpression, cells were transfected with pcDNA3 (Invitrogen), pcDNA3-DcR3 (generated as outlined below), myrAkt δ4 (Addgene Plasmid 10841) [62] and NFATc1 (Addgene Plasmid 11788) [63] both obtained from Addgene (Cambridge, MA, USA), using Lipofectamine 2000 (Invitrogen). The pcDNA3-DcR3 plasmid was generated by PCR from the clone pENTR223-TNFRSF6B, provided by the ORFeome Collaboration via the Genomics and Proteomics Core Facility (DKFZ, Heidelberg, Germany) using the following forward (F) and reverse (R) primers containing BamHI and EcoRI restrictions sites: 5′-GATCATGTAGAGGATCCACCATGAGGGCGCTGGAGGGG-3′ (F) and 5′-ATACTGCTACGAATTCTCAGTGCACAGGGAGGAAGC-3′ (R). The PCR products were digested with BamHI and EcoRI and cloned into the BamHI and EcoRI sites of pcDNA3 (Invitrogen). For the generation of stable transfectants, complete medium containing Geneticin® (G418, Invitrogen) at a concentration of 1.5 mg/mL was used to select stably transfected cells.

### Preparation and culture of tissue slices and immunohistochemistry

The usage of tumor tissue for research purposes was approved by the local ethics committee of the University Hospital of Heidelberg, Germany. All data were analyzed anonymously. Written informed consent from the donors or the next of kin was obtained for use of these samples in research. Fresh human renal cell carcinoma tissue samples were obtained from the Tissue Bank of the Center for National Tumor Diseases (NCT, Heidelberg, Germany) directly after surgery and maintained in DMEM medium on ice. Tissue samples were cut into 300 μm thick slices by a Leica VT1200 S vibrating blade microtome (Leica, Wetzlar, Germany). Slices were then placed on porous filter membrane inserts in six-well plates and cultured in DMEM supplemented with penicillin (100 U/mL) and streptomycin (100 mg/mL) (Sigma Aldrich) in a conventional CO_2_ incubator. After 24 hours, slices were treated with LY294002 for further 24 h. After treatment, tissue slices were fixed in 10% neutral-buffered formalin and embedded in paraffin. Four micrometer sections were stained with H&E or subjected to immunohistochemistry.

Paraffin-embedded tissue sections were dewaxed and rehydrated using xylene and a series of graded alcohols, followed by heat-induced antigen retrieval with a target retrieval solution (S2301, DakoCytomation, Glostrup, Denmark) in a pressure cooker for 15 min. For staining an automated staining system (Techmate 500, DakoCytomation) with avidin-biotin-complex peroxidase technique using aminoethylcarbazole for visualization and hematoxylin for counterstaining was used. Sections were incubated with primary antibody (anti-DcR3; BioLegend; London, United Kingdom; #333202; 6.25 μg/mL) for 30 min at room temperature and processed according to manufacturer’s protocol for the following kits: ChemMate Detection Kit (K5003, DakoCytomation), ChemMate Buffer Kit (K5006, DakoCytomation), Avidin/Biotin Blocking Kit (SP-2001, Vector Laboratories, Burlingame, CA, USA). For negative control of the staining procedure, primary antibody was omitted with all other experimental conditions kept constant.

### Reporter gene assays

Cells were seeded into 12-well dishes and co-transfected with Renilla luciferase pRL-SV40P (Promega, Madison, WI, USA) and FHRE-Luc (Addgene Plasmid 1789) [64] or pGL3-NFAT luciferase (Addgene Plasmid 17870) [65]. 24 h after transfection cells were subjected to LY294002 or AKT-inhibitor IV treatment for further 24 h prior to the preparation of cell lysates. Both Firefly and Renilla luciferase activities were quantified using the dual-luciferase-reporter assay system (Promega), according to the manufacturer’s instructions.

### Quantitative real-time PCR

Quantitative real-time PCR was performed as described previously [66]. Following primer pairs were used:

DcR3: 5′-CCACTACACGCAGTTCTGGA-3′ (forward)

5′-GTGCTCCAAGCAGAAACCAG-3′ (reverse)

MMP-2: 5′-CTTCCAAGTCTGGAGCGATGT-3′ (forward)

5′-TACCGTCAAAGGGGTATCCAT-3′ (reverse)

MMP-7: 5′-GAGTGCCAGATGTTGCAGAA-3′ (forward)

5′-AAATGCAGGGGGATCTCTTT-3′ (reverse)

MMP-9: 5′-TTGACAGCGACAAGAAGTGG-3′ (forward)

5′-ACATAGGGTACATGAGCGCC-3′ (reverse)

integrin alpha 4: 5′-GAGTGCAATGCAGACCTTGA-3′ (forward)

5′-GGCCTTCCAGTTGGATATGA-3′ (reverse)

integrin beta 1: 5′-AACTGCACCAGCCCATTTAG-3′ (forward)

5′-ACATTCCTCCAGCCAATCAG-3′ (reverse)

uPA: 5′-GCCATCCCGGACTATACAGA-3′ (forward)

5′-ACACAGCATTTTGGTGGTGA-3′ (reverse)

18S: 5′-CATGGCCGTTCTTAGTTGGT-3′ (forward)

5′-ATGCCAGAGTCTCGTTCGTT-3′ (reverse)

### Immunoblot analysis

Cells were rinsed with ice-cold PBS and lysed with lysis buffer (120 mM NaCl, 50 mM Tris–HCl (pH 8.0), 5 mM EDTA, 0.5% Triton X-100) containing phenylmethylsulfonyl-flouride (1 mM), proteinase inhibitors (Roche, Mannheim, Germany, #1697498) and phosphatase inhibitors (25 mM NaF, 200 μM NaVO_3_, 10 mM NaPP_i_). After 15 min incubation on ice, lysates were centrifuged at 16 000 *g* for 20 min. For cytoplasmic and nuclear fractions cells were harvested and processed with the Nuclear Extraction Kit (Active Motif, Rixensart, Belgium) according to manufacturer’s protocol. For protein isolation from human tissue, frozen tissue samples kindly provided by the Tissue Bank of the Center for National Tumor Diseases (NCT, Heidelberg, Germany) were suspended in 100 μl lysis buffer and shock frozen in liquid nitrogen. Thereafter 5 mm grinding balls were added. The tissue samples were homogenized by the use of a Mixer Mill MM 200 (Retsch, Haan, Germany) and centrifuged for 10 min at 16 000 *g*. For the isolation of proteins from supernatant, cells were incubated in serum free medium. After 24–48 h the medium was harvested and centrifuged for 5 min at 1000 *g*. 200 μL of the supernatant were used for precipitation [67]. A Ponceau S-stained protein band was used for normalization. Total protein concentration was measured by Bradford-Assay (Bio-Rad, Munich, Germany). 20–40 μg protein was separated on 10–15% polyacrylamide gels and blotted onto nitrocellulose (Bio-Rad) by standard procedures. Membranes were washed, incubated with primary antibody over night, washed again incubated with secondary antibody (1:3000) (horseradish peroxidase-conjugated, Bio-Rad) and visualized by an enhanced chemiluminescence detection system (GE Healthcare, Munich, Germany). Following primary antibodies were used: anti-DcR3 (BioLegend, #333202), anti-β-actin (Merck Millipore, Darmstadt, Germany, #mab1501), anti-AKT (Cell Signaling, Danvers, MA, USA, #9272), anti-phospho-AKT (Cell Signaling, #4058), anti-P70S6K (Cell Signaling, #9202), anti-phospho-P70S6K (Cell Signaling, #9206), anti-GSK-3β (Cell Signaling, #9315), anti-phospho-GSK-3β (Cell Signaling, #9336), anti-PARP (BD Pharmingen, San Diego, CA, USA, #556362), anti-Caspase 3 (Imgenex, San Diego, CA, USA, #IMG-144A), anti-NFATc1 (Abcam, Cambridge, MA, USA, #ab25916), and anti-ITGA4 (GeneTex, Irvine, CA, USA, #GTX61691).

### Scratch motility assay

Cells (5 × 10^5^) were seeded into 12–well culture dishes. 24 h thereafter, a 100 μm scratch was placed within the confluent monolayer with a pipette tip. Cells were then placed into the incubation chamber of an Olympus IX81 microscope and cultivated at 37°C, 40% humidity and 5% CO_2_. Throughout 24 h pictures were taken at intervals of 1–2 h with an Olympus U-CMAD3 camera by CellR software. Migratory activity was calculated with Image J software, based on the cell-free areas.

### Proliferation and clonogenicity assay

For the assessment of proliferation, 2.5 × 10^5^ cells were seeded into 6-cm culture dishes and counted after 24 h, 48 h and 72 h using the trypan blue exclusion assay. For clonogenicity assays, 500 cells were seeded into 6–well culture dishes and incubated for seven days prior to crystal violet staining and colony counting.

### Boyden chamber assay

The invasive capacity of cells was analyzed by the use of Matrigel™ coated chambers (BD Bioscience, Sparks, MD, USA) according to manufacturer’s protocol. Briefly, cells were starved for 24 h with serum free medium. After trypsinization, 10^5^ cells, suspended in 0.5 mL serum free medium, were seeded into the boyden chamber insert. 0.75 mL medium enriched with 20% FCS was added as a chemoattractant into the well. Cells were allowed to invade the Matrigel™ matrix for 24–48 h. Thereafter, transmigrated cells were fixed and stained with crystal violet.

### Spheroid invasion assay

Cells (75 000–100 000) resuspended in 20 μL medium were suspended on the lid of a 100-mm Petri dish to form spheroids. 48 h later, spheroids were placed in cell culture dishes, coated with 2% sterile agar (Merck)/PBS (solidified) and filled with growth medium. 24 h later, spheroids were embedded into collagen gels. Collagen gels were made by mixing Vitrogen (97% collagen type I; Nutacon BV, Leimuiden, the Netherlands) with 10-fold concentrated minimal essential medium (Life Technologies) and sterile 0.1 M sodium hydroxide, resulting in a final concentration of 2.4 mg/mL collagen. Collagen solution was distributed into 24-well plates (0.5 mL/well) which had previously been coated with 2% sterile agar (Merck)/PBS (solidified). After solidification gels were overlaid with growth medium. Cell invasion was monitored at intervals of 24 h by photographing spheroids with an inverted Olympus IMT2-RFA/340 phase contrast microscope.

### Cytotoxicity assay

For acute cytotoxicity assays, 10^4^ cells were plated in 96-well plates, adhered for 24 h, and exposed to CD95L for 24 h. The percentage of surviving cells was assessed by staining with crystal violet [68]. Briefly, the supernatant was removed, and the cells were incubated in a 2% crystal violet solution in 20% methanol for 10 min. The plates were washed in running tap water and air-dried for 24 h. Crystal violet was solubilized by the addition of a 0.1 M sodium citrate buffer in 50% ethanol. The absorption was measured at 550 nm using a microplate reader (Bio-Rad).

### Statistical methods

For all data, significant differences were identified using the unpaired 2-sided Student *t* test. Throughout, *P* values < 0.05 were considered significant and are indicated as follows: *P < 0.05, **P < 0.01, ***P < 0.001.

## Competing interests

The authors declared that they have no competing interests.

## Authors’ contributions

DW carried out the experiments, participated in study conception and design, analyzed and interpreted the data, and was involved in drafting the manuscript. KET participated in study conception and design, analyzed and interpreted the data, and was involved in drafting the manuscript. SMG participated in study conception and design, and revised the manuscript for important intellectual content. AH participated in study conception and design, and revised the manuscript for important intellectual content. NW participated in study conception and design, and revised the manuscript for important intellectual content. WR participated in study conception and design, analyzed and interpreted the data, and was involved in drafting the manuscript. All authors read and approved the final manuscript.

## Supplementary Material

Additional file 1: Figure S1 DcR3 expression in RCC. **(A)** Quantitative real-time-PCR assaying relative DcR3 mRNA expression levels. Expression data were normalized to internal 18S rRNA expression. **(B)** Cytotoxicity assay of ACHN and 769-P cells transfected with two different DcR3-specific siRNAs or a non-specific siRNA (scram). Cells were treated with CD95L (200 ng/μL) for 24 h prior to crystal violet staining (mean ± SEM; n=3; **p<0.01; scram vs. DcR3-si1 and scram vs. DcR3-si2; T-test). **(C)** Cytotoxicity assay of CD95L sensitive LN18 glioblastoma cells stably overexpressing DcR3 or an empty vector control (neo). Cells were treated with CD95L (200 ng/μL) for 24 h prior to crystal violet staining (mean ± SEM; n=3; *p<0.05; T-test). Since ACHN and 769-P RCC cells are resistant to CD95L induced cytotoxicity, CD95L sensitive LN18 glioblastoma cells were chosen to analyze the protective effect of DcR3 overexpression. **(D)** Scratch motility assay of ACHN and 769-P cells transfected with two different DcR3-specific siRNAs or a non-specific siRNA (scram). Migration was measured over a time course of 24 h. Representative images are shown (magnification: 100×). **(E)** Scratch motility assay of ACHN and 769-P stably overexpressing DcR3 or an empty control vector (neo). Migration was measured over a time course of 12 h (ACHN) or 24 h (769-P). Representative images are shown (magnification: 100×). Click here for file

Additional file 2: Figure S2 DcR3 expression does not affect proliferation or clonogenicity in RCC. **(A)** Proliferation assay of ACHN and 769-P cells 24, 48 and 72 h after transfection with two different DcR3-specific siRNAs or a non-specific siRNA (scram). Cells were trypsinized and counted (mean ± SEM; n=3). **(B)** Proliferation assay of ACHN and 769-P cells stably overexpressing DcR3 or an empty vector control (neo) after 24, 48 and 72 h. Cells were trypsinized and counted (mean ± SEM; n=3). **(C)** Clonogenicity assay of ACHN and 769-P cells transfected with two different DcR3-specific siRNAs or a non-specific siRNA (scram). Cells were grown for 7–9 days, subsequently stained with crystal violet and colonies were counted (mean ± SEM; n=3). **(D)** Clonogenicity assay of ACHN and 769-P cells stably overexpressing DcR3 or an empty vector control (neo). Cells were grown for 7–9 days, subsequently stained with crystal violet and colonies were counted (mean ± SEM; n=3). Click here for file

Additional file 3: Figure S3 NFAT mediated DcR3 expression is PI3K/AKT dependent. **(A)** Relative Forkhead response element–luciferase reporter activity of ACHN and 769-P cells 24 h after treatment with LY294002 (50 μM) or AKT-inhibitor IV (10 μM) (mean ± SEM; n=3; *p<0.05; T-test). **(B)** Relative Forkhead response element–luciferase reporter activity of ACHN and 769-P cells 24 h post transfection with myrAKT or an empty vector control (neo) (mean ± SEM; n=3; **p<0.01, ***p<0.001; T-test). **(C)** Representative immunoblot analysis of whole-cell lysates of *ex vivo* tissue 48 h after treatment with LY294002 (100 μM). Click here for file

## References

[B1] Cohen HT McGovern FJ Renal-cell carcinoma N Engl J Med 2005 353 2477 2490 10.1056/NEJMra043172 16339096

[B2] Schmidinger M Bellmunt J Plethora of agents, plethora of targets, plethora of side effects in metastatic renal cell carcinoma Cancer Treat Rev 2010 36 416 424 10.1016/j.ctrv.2010.01.003 20163917

[B3] Motzer RJ Russo P Systemic therapy for renal cell carcinoma J Urol 2000 163 408 417 10.1016/S0022-5347(05)67889-5 10647643

[B4] Iliopoulos O Levy AP Jiang C Kaelin WG Jr Goldberg MA Negative regulation of hypoxia-inducible genes by the von Hippel-Lindau protein Proc Natl Acad Sci U S A 1996 93 10595 10599 10.1073/pnas.93.20.10595 8855223 PMC38198

[B5] Tostain J Li G Gentil-Perret A Gigante M Carbonic anhydrase 9 in clear cell renal cell carcinoma: a marker for diagnosis, prognosis and treatment Eur J Cancer 2010 46 3141 3148 10.1016/j.ejca.2010.07.020 20709527

[B6] Kaelin WG Jr The von Hippel-Lindau tumor suppressor protein and clear cell renal carcinoma Clin Cancer Res 2007 13 680s 684s 10.1158/1078-0432.CCR-06-1865 17255293

[B7] Cho D Signoretti S Regan M Mier JW Atkins MB The role of mammalian target of rapamycin inhibitors in the treatment of advanced renal cancer Clin Cancer Res 2007 13 758s 763s 10.1158/1078-0432.CCR-06-1986 17255306

[B8] Pitti RM Marsters SA Lawrence DA Roy M Kischkel FC Dowd P Huang A Donahue CJ Sherwood SW Baldwin DT Godowski PJ Wood WI Gurney AL Hillan KJ Cohen RL Goddard AD Botstein D Ashkenazi A Genomic amplification of a decoy receptor for Fas ligand in lung and colon cancer Nature 1998 396 699 703 10.1038/25387 9872321

[B9] Roth W Isenmann S Nakamura M Platten M Wick W Kleihues P Bahr M Ohgaki H Ashkenazi A Weller M Soluble decoy receptor 3 is expressed by malignant gliomas and suppresses CD95 ligand-induced apoptosis and chemotaxis Cancer Res 2001 61 2759 2765 11289159

[B10] Yang CR Hsieh SL Teng CM Ho FM Su WL Lin WW Soluble decoy receptor 3 induces angiogenesis by neutralization of TL1A, a cytokine belonging to tumor necrosis factor superfamily and exhibiting angiostatic action Cancer Res 2004 64 1122 1129 10.1158/0008-5472.CAN-03-0609 14871847

[B11] Migone TS Zhang J Luo X Zhuang L Chen C Hu B Hong JS Perry JW Chen SF Zhou JX Cho YH Ullrich S Kanakaraj P Carrell J Boyd E Olsen HS Hu G Pukac L Liu D Ni J Kim S Gentz R Feng P Moore PA Ruben SM Wei P TL1A is a TNF-like ligand for DR3 and TR6/DcR3 and functions as a T cell costimulator Immunity 2002 16 479 492 10.1016/S1074-7613(02)00283-2 11911831

[B12] Yu KY Kwon B Ni J Zhai Y Ebner R Kwon BS A newly identified member of tumor necrosis factor receptor superfamily (TR6) suppresses LIGHT-mediated apoptosis J Biol Chem 1999 274 13733 13736 10.1074/jbc.274.20.13733 10318773

[B13] Shi G Luo H Wan X Salcedo TW Zhang J Wu J Mouse T cells receive costimulatory signals from LIGHT, a TNF family member Blood 2002 100 3279 3286 10.1182/blood-2002-05-1404 12384428

[B14] Chang YC Hsu TL Lin HH Chio CC Chiu AW Chen NJ Lin CH Hsieh SL Modulation of macrophage differentiation and activation by decoy receptor 3 J Leukoc Biol 2004 75 486 494 14657214 10.1189/jlb.0903448

[B15] Yang CR Wang JH Hsieh SL Wang SM Hsu TL Lin WW Decoy receptor 3 (DcR3) induces osteoclast formation from monocyte/macrophage lineage precursor cells Cell Death Differ 2004 11 Suppl 1 S97 S107 15002040 10.1038/sj.cdd.4401403

[B16] Bai C Connolly B Metzker ML Hilliard CA Liu X Sandig V Soderman A Galloway SM Liu Q Austin CP Caskey CT Overexpression of M68/DcR3 in human gastrointestinal tract tumors independent of gene amplification and its location in a four-gene cluster Proc Natl Acad Sci U S A 2000 97 1230 1235 10.1073/pnas.97.3.1230 10655513 PMC15578

[B17] Tsuji S Hosotani R Yonehara S Masui T Tulachan SS Nakajima S Kobayashi H Koizumi M Toyoda E Ito D Kami K Mori T Fujimoto K Doi R Imamura M Endogenous decoy receptor 3 blocks the growth inhibition signals mediated by Fas ligand in human pancreatic adenocarcinoma Int J Cancer 2003 106 17 25 10.1002/ijc.11170 12794752

[B18] Wu Y Han B Sheng H Lin M Moore PA Zhang J Wu J Clinical significance of detecting elevated serum DcR3/TR6/M68 in malignant tumor patients Int J Cancer 2003 105 724 732 10.1002/ijc.11138 12740925

[B19] Macher-Goeppinger S Aulmann S Wagener N Funke B Tagscherer KE Haferkamp A Hohenfellner M Kim S Autschbach F Schirmacher P Roth W Decoy receptor 3 is a prognostic factor in renal cell cancer Neoplasia 2008 10 1049 1056 18813347 10.1593/neo.08626 PMC2546583

[B20] Chang PM Chen PM Hsieh SL Tzeng CH Liu JH Chiou TJ Wang WS Yen CC Gau JP Yang MH Expression of a soluble decoy receptor 3 in patients with diffuse large B-cell lymphoma predicts clinical outcome Int J Oncol 2008 33 549 554 18695885

[B21] Takahama Y Yamada Y Emoto K Fujimoto H Takayama T Ueno M Uchida H Hirao S Mizuno T Nakajima Y The prognostic significance of overexpression of the decoy receptor for Fas ligand (DcR3) in patients with gastric carcinomas Gastric Cancer 2002 5 61 68 10.1007/s101200200011 12111580

[B22] Lohi J Tani T Laitinen L Kangas L Lehto VP Virtanen I Tenascin and fibronectin isoforms in human renal cell carcinomas, renal cell carcinoma cell lines and xenografts in nude mice Int J Cancer 1995 63 442 449 10.1002/ijc.2910630324 7591246

[B23] Kaplan RN Riba RD Zacharoulis S Bramley AH Vincent L Costa C MacDonald DD Jin DK Shido K Kerns SA Zhu Z Hicklin D Wu Y Port JL Altorki N Port ER Ruggero D Shmelkov SV Jensen KK Rafii S Lyden D VEGFR1-positive haematopoietic bone marrow progenitors initiate the pre-metastatic niche Nature 2005 438 820 827 10.1038/nature04186 16341007 PMC2945882

[B24] Porta C Figlin RA Phosphatidylinositol-3-kinase/Akt signaling pathway and kidney cancer, and the therapeutic potential of phosphatidylinositol-3-kinase/Akt inhibitors J Urol 2009 182 2569 2577 10.1016/j.juro.2009.08.085 19836781

[B25] Macian F NFAT proteins: key regulators of T-cell development and function Nat Rev Immunol 2005 5 472 484 10.1038/nri1632 15928679

[B26] Ho CH Chen CL Li WY Chen CJ Decoy receptor 3, upregulated by Epstein-Barr virus latent membrane protein 1, enhances nasopharyngeal carcinoma cell migration and invasion Carcinogenesis 2009 30 1443 1451 10.1093/carcin/bgp135 19483191

[B27] Ge Z Sanders AJ Ye L Wang Y Jiang WG Expression of death decoy receptor-3 (DcR3) in human breast cancer and its functional effects on breast cancer cells in vitro J Exp Ther Oncol 2011 9 109 118 21699018

[B28] Connor JP Felder M Kapur A Onujiogu N DcR3 binds to ovarian cancer via heparan sulfate proteoglycans and modulates tumor cells response to platinum with corresponding alteration in the expression of BRCA1 BMC Cancer 2012 12 176 10.1186/1471-2407-12-176 22583667 PMC3462721

[B29] Tateishi K Miura Y Hayashi S Takahashi M Kurosaka M DcR3 protects THP-1 macrophages from apoptosis by increasing integrin alpha4 Biochem Biophys Res Commun 2009 389 593 598 10.1016/j.bbrc.2009.09.027 19748482

[B30] Markovic-Lipkovski J Brasanac D Muller GA Muller CA Cadherins and integrins in renal cell carcinoma: an immunohistochemical study Tumori 2001 87 173 178 11504373 10.1177/030089160108700312

[B31] Hartmann TN Burger JA Glodek A Fujii N Burger M CXCR4 chemokine receptor and integrin signaling co-operate in mediating adhesion and chemoresistance in small cell lung cancer (SCLC) cells Oncogene 2005 24 4462 4471 10.1038/sj.onc.1208621 15806155

[B32] Rebhun RB Cheng H Gershenwald JE Fan D Fidler IJ Langley RR Constitutive expression of the alpha4 integrin correlates with tumorigenicity and lymph node metastasis of the B16 murine melanoma Neoplasia 2010 12 173 182 20126475 10.1593/neo.91604 PMC2814355

[B33] Yang CR Hsieh SL Ho FM Lin WW Decoy receptor 3 increases monocyte adhesion to endothelial cells via NF-kappa B-dependent up-regulation of intercellular adhesion molecule-1, VCAM-1, and IL-8 expression J Immunol 2005 174 1647 1656 15661928 10.4049/jimmunol.174.3.1647

[B34] Song K Zhu F Zhang HZ Shang ZJ Tumor necrosis factor-alpha enhanced fusions between oral squamous cell carcinoma cells and endothelial cells via VCAM-1/VLA-4 pathway Exp Cell Res 2012 318 1707 1715 10.1016/j.yexcr.2012.05.022 22664325

[B35] Miyata Y Iwata T Ohba K Kanda S Nishikido M Kanetake H Expression of matrix metalloproteinase-7 on cancer cells and tissue endothelial cells in renal cell carcinoma: prognostic implications and clinical significance for invasion and metastasis Clin Cancer Res 2006 12 6998 7003 10.1158/1078-0432.CCR-06-1626 17145820

[B36] Paule B Deslandes E Le Mouel SP Bastien L Podgorniak MP Allory Y de la Taille A Menashi S Calvo F Mourah S Identification of a novel biomarker signature associated with risk for bone metastasis in patients with renal cell carcinoma Int J Biol Markers 2010 25 112 115 20544684 10.1177/172460081002500209

[B37] Chang YC Chan YH Jackson DG Hsieh SL The glycosaminoglycan-binding domain of decoy receptor 3 is essential for induction of monocyte adhesion J Immunol 2006 176 173 180 16365408 10.4049/jimmunol.176.1.173

[B38] Bourguignon LY Zhu H Shao L Chen YW CD44 interaction with c-Src kinase promotes cortactin-mediated cytoskeleton function and hyaluronic acid-dependent ovarian tumor cell migration J Biol Chem 2001 276 7327 7336 10.1074/jbc.M006498200 11084024

[B39] Bourguignon LY Zhu H Zhou B Diedrich F Singleton PA Hung MC Hyaluronan promotes CD44v3-Vav2 interaction with Grb2-p185(HER2) and induces Rac1 and Ras signaling during ovarian tumor cell migration and growth J Biol Chem 2001 276 48679 48692 10.1074/jbc.M106759200 11606575

[B40] De Oliveira T Abiatari I Raulefs S Sauliunaite D Erkan M Kong B Friess H Michalski CW Kleeff J Syndecan-2 promotes perineural invasion and cooperates with K-ras to induce an invasive pancreatic cancer cell phenotype Mol Cancer 2012 11 19 10.1186/1476-4598-11-19 22471946 PMC3350462

[B41] Hsu MJ Lin WW Tsao WC Chang YC Hsu TL Chiu AW Chio CC Hsieh SL Enhanced adhesion of monocytes via reverse signaling triggered by decoy receptor 3 Exp Cell Res 2004 292 241 251 10.1016/j.yexcr.2003.09.019 14697332

[B42] Yu CR Lee YS Mahdi RM Surendran N Egwuagu CE Therapeutic targeting of STAT3 (signal transducers and activators of transcription 3) pathway inhibits experimental autoimmune uveitis PLoS One 2012 7 e29742 10.1371/journal.pone.0029742 22238646 PMC3252323

[B43] Fukuda A Wang SC Morris JP Folias AE Liou A Kim GE Akira S Boucher KM Firpo MA Mulvihill SJ Hebrok M Stat3 and MMP7 contribute to pancreatic ductal adenocarcinoma initiation and progression Cancer Cell 2011 19 441 455 10.1016/j.ccr.2011.03.002 21481787 PMC3075548

[B44] Chen PH Yang CR Decoy receptor 3 expression in AsPC-1 human pancreatic adenocarcinoma cells via the phosphatidylinositol 3-kinase-, Akt-, and NF-kappa B-dependent pathway J Immunol 2008 181 8441 8449 19050262 10.4049/jimmunol.181.12.8441

[B45] Ho CH Hsu CF Fong PF Tai SK Hsieh SL Chen CJ Epstein-Barr virus transcription activator Rta upregulates decoy receptor 3 expression by binding to its promoter J Virol 2007 81 4837 4847 10.1128/JVI.02448-06 17301127 PMC1900157

[B46] Ni YG Berenji K Wang N Oh M Sachan N Dey A Cheng J Lu G Morris DJ Castrillon DH Gerard RD Rothermel BA Hill JA Foxo transcription factors blunt cardiac hypertrophy by inhibiting calcineurin signaling Circulation 2006 114 1159 1168 10.1161/CIRCULATIONAHA.106.637124 16952979 PMC4118290

[B47] Cai T Li X Ding J Luo W Li J Huang C A cross-talk between NFAT and NF-kappaB pathways is crucial for nickel-induced COX-2 expression in Beas-2B cells Curr Cancer Drug Targets 2011 11 548 559 10.2174/156800911795656001 21486220 PMC3759234

[B48] Liu Q Chen Y Auger-Messier M Molkentin JD Interaction between NFkappaB and NFAT coordinates cardiac hypertrophy and pathological remodeling Circ Res 2012 110 1077 1086 10.1161/CIRCRESAHA.111.260729 22403241 PMC3341669

[B49] Oeckinghaus A Hayden MS Ghosh S Crosstalk in NF-kappaB signaling pathways Nat Immunol 2011 12 695 708 21772278 10.1038/ni.2065

[B50] Lawrence MC Naziruddin B Levy MF Jackson A McGlynn K Calcineurin/nuclear factor of activated T cells and MAPK signaling induce TNF-{alpha} gene expression in pancreatic islet endocrine cells J Biol Chem 2011 286 1025 1036 10.1074/jbc.M110.158675 21059644 PMC3020709

[B51] Lee HL Bae OY Baek KH Kwon A Hwang HR Qadir AS Park HJ Woo KM Ryoo HM Baek JH High extracellular calcium-induced NFATc3 regulates the expression of receptor activator of NF-kappaB ligand in osteoblasts Bone 2011 49 242 249 10.1016/j.bone.2011.04.006 21514407

[B52] Tsai EY Falvo JV Tsytsykova AV Barczak AK Reimold AM Glimcher LH Fenton MJ Gordon DC Dunn IF Goldfeld AE A lipopolysaccharide-specific enhancer complex involving Ets, Elk-1, Sp1, and CREB binding protein and p300 is recruited to the tumor necrosis factor alpha promoter in vivo Mol Cell Biol 2000 20 6084 6094 10.1128/MCB.20.16.6084-6094.2000 10913190 PMC86084

[B53] Wang Q Ji Y Wang X Evers BM Isolation and molecular characterization of the 5′-upstream region of the human TRAIL gene Biochem Biophys Res Commun 2000 276 466 471 10.1006/bbrc.2000.3512 11027498

[B54] Xiao S Matsui K Fine A Zhu B Marshak-Rothstein A Widom RL Ju ST FasL promoter activation by IL-2 through SP1 and NFAT but not Egr-2 and Egr-3 Eur J Immunol 1999 29 3456 3465 10.1002/(SICI)1521-4141(199911)29:11<3456::AID-IMMU3456>3.0.CO;2-B 10556800

[B55] Yoshida T Sakai T Promoter of TRAIL-R2 gene Vitam Horm 2004 67 35 49 15110170 10.1016/S0083-6729(04)67003-8

[B56] Garlich JR De P Dey N Su JD Peng X Miller A Murali R Lu Y Mills GB Kundra V Shu HK Peng Q Durden DL A vascular targeted pan phosphoinositide 3-kinase inhibitor prodrug, SF1126, with antitumor and antiangiogenic activity Cancer Res 2008 68 206 215 10.1158/0008-5472.CAN-07-0669 18172313

[B57] Mahadevan D Chiorean EG Harris WB Von Hoff DD Stejskal-Barnett A Qi W Anthony SP Younger AE Rensvold DM Cordova F Shelton CF Becker MD Garlich JR Durden DL Ramanathan RK Phase I pharmacokinetic and pharmacodynamic study of the pan-PI3K/mTORC vascular targeted pro-drug SF1126 in patients with advanced solid tumours and B-cell malignancies Eur J Cancer 2012 48 3319 3327 10.1016/j.ejca.2012.06.027 22921184 PMC3826796

[B58] Gharbi SI Zvelebil MJ Shuttleworth SJ Hancox T Saghir N Timms JF Waterfield MD Exploring the specificity of the PI3K family inhibitor LY294002 Biochem J 2007 404 15 21 10.1042/BJ20061489 17302559 PMC1868829

[B59] Engelman JA Targeting PI3K signalling in cancer: opportunities, challenges and limitations Nat Rev Cancer 2009 9 550 562 10.1038/nrc2664 19629070

[B60] Merseburger AS Hennenlotter J Kuehs U Simon P Kruck S Koch E Stenzl A Kuczyk MA Activation of PI3K is associated with reduced survival in renal cell carcinoma Urol Int 2008 80 372 377 10.1159/000132694 18587247

[B61] Schmitt M Pawlita M High-throughput detection and multiplex identification of cell contaminations Nucleic Acids Res 2009 37 e119 10.1093/nar/gkp581 19589807 PMC2764421

[B62] Kohn AD Takeuchi F Roth RA Akt, a pleckstrin homology domain containing kinase, is activated primarily by phosphorylation J Biol Chem 1996 271 21920 21926 10.1074/jbc.271.36.21920 8702995

[B63] Northrop JP Ho SN Chen L Thomas DJ Timmerman LA Nolan GP Admon A Crabtree GR NF-AT components define a family of transcription factors targeted in T-cell activation Nature 1994 369 497 502 10.1038/369497a0 8202141

[B64] Brunet A Bonni A Zigmond MJ Lin MZ Juo P Hu LS Anderson MJ Arden KC Blenis J Greenberg ME Akt promotes cell survival by phosphorylating and inhibiting a Forkhead transcription factor Cell 1999 96 857 868 10.1016/S0092-8674(00)80595-4 10102273

[B65] Clipstone NA Crabtree GR Identification of calcineurin as a key signalling enzyme in T-lymphocyte activation Nature 1992 357 695 697 10.1038/357695a0 1377362

[B66] Fassl A Tagscherer KE Richter J Berriel Diaz M Alcantara Llaguno SR Campos B Kopitz J Herold-Mende C Herzig S Schmidt MH Parada LF Wiestler OD Roth W Notch1 signaling promotes survival of glioblastoma cells via EGFR-mediated induction of anti-apoptotic Mcl-1 Oncogene 2012 31 4698 4708 10.1038/onc.2011.615 22249262

[B67] Wessel D Flugge UI A method for the quantitative recovery of protein in dilute solution in the presence of detergents and lipids Anal Biochem 1984 138 141 143 10.1016/0003-2697(84)90782-6 6731838

[B68] Gillies RJ Didier N Denton M Determination of cell number in monolayer cultures Anal Biochem 1986 159 109 113 10.1016/0003-2697(86)90314-3 3812988

