# Supplementary figures and images for: The soluble Decoy Receptor 3 is regulated by a PI3K-dependent mechanism and promotes migration and invasion in renal cell carcinoma
Source: Mol Cancer. 2013 Oct 10;12:120. doi: 10.1186/1476-4598-12-120 (PMC3852559; doi:10.1186/1476-4598-12-120)

# Supplementary Figure 1

A

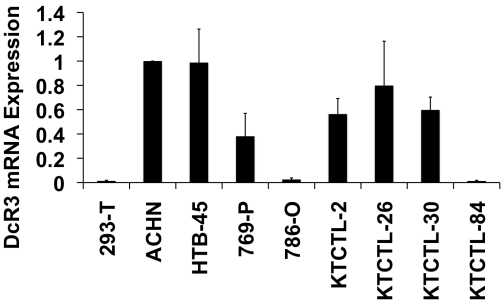

B

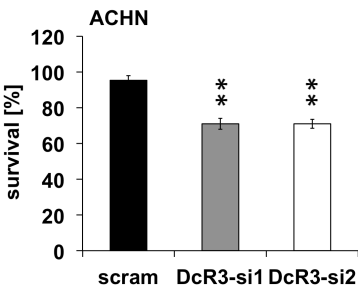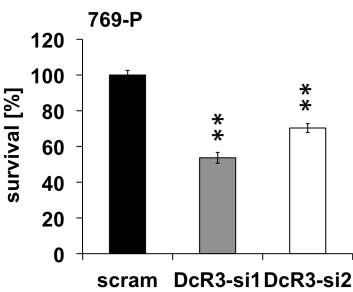

C

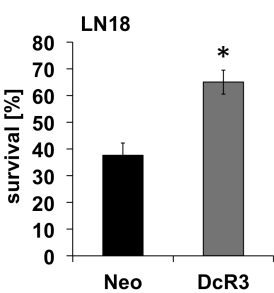

D

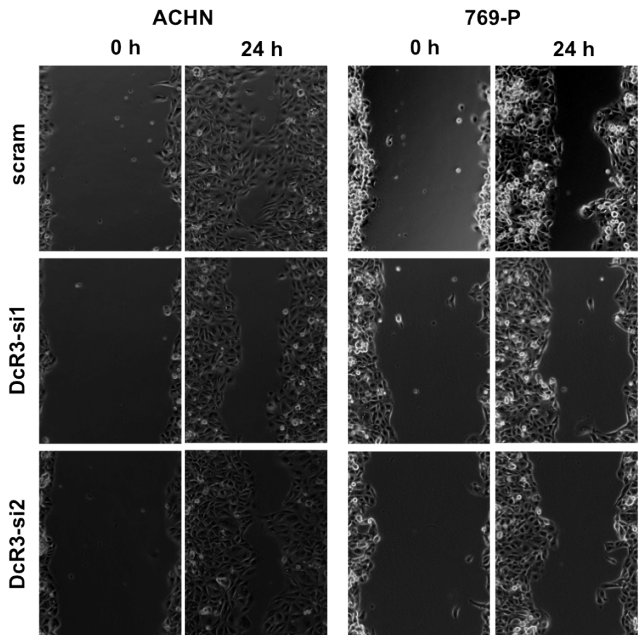

E

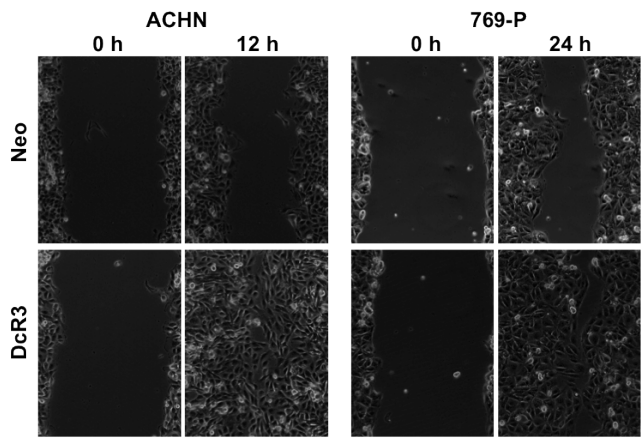

Supplement: Additional file 1: Figure S1 — DcR3 expression in RCC. (A) Quantitative real-time-PCR assaying relative DcR3 mRNA expression levels. Expression data were normalized to internal 18S rRNA expression. (B) Cytotoxicity assay of ACHN and 769-P cells transfected with two different DcR3-specific siRNAs or a non-specific siRNA (scram). Cells were treated with CD95L (200 ng/μL) for 24 h prior to crystal violet staining (mean ± SEM; n=3; **p<0.01; scram vs. DcR3-si1 and scram vs. DcR3-si2; T-test). (C) Cytotoxicity assay of CD95L sensitive LN18 glioblastoma cells stably overexpressing DcR3 or an empty vector control (neo). Cells were treated with CD95L (200 ng/μL) for 24 h prior to crystal violet staining (mean ± SEM; n=3; *p<0.05; T-test). Since ACHN and 769-P RCC cells are resistant to CD95L induced cytotoxicity, CD95L sensitive LN18 glioblastoma cells were chosen to analyze the protective effect of DcR3 overexpression. (D) Scratch motility assay of ACHN and 769-P cells transfected with two different DcR3-specific siRNAs or a non-specific siRNA (scram). Migration was measured over a time course of 24 h. Representative images are shown (magnification: 100×). (E) Scratch motility assay of ACHN and 769-P stably overexpressing DcR3 or an empty control vector (neo). Migration was measured over a time course of 12 h (ACHN) or 24 h (769-P). Representative images are shown (magnification: 100×). [file 1476-4598-12-120-S1.pdf]

# Supplementary Figure 2

A

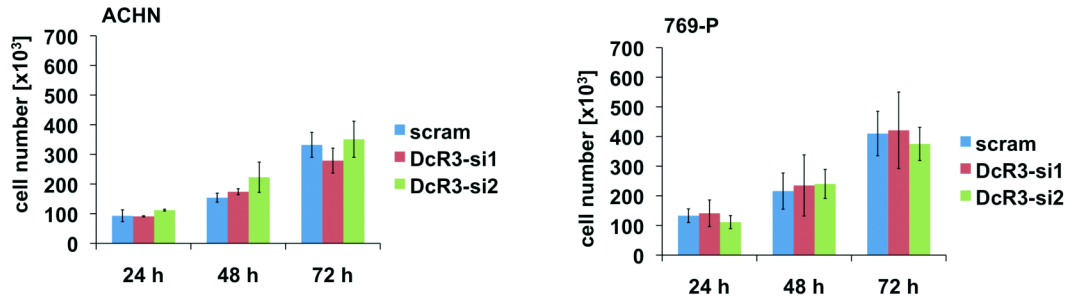

B

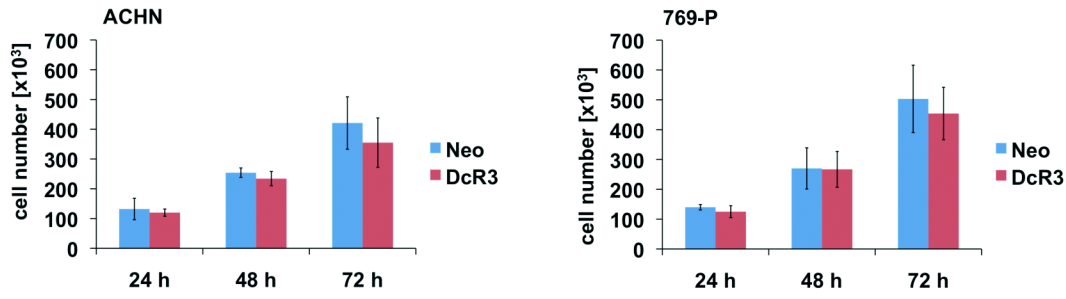

C

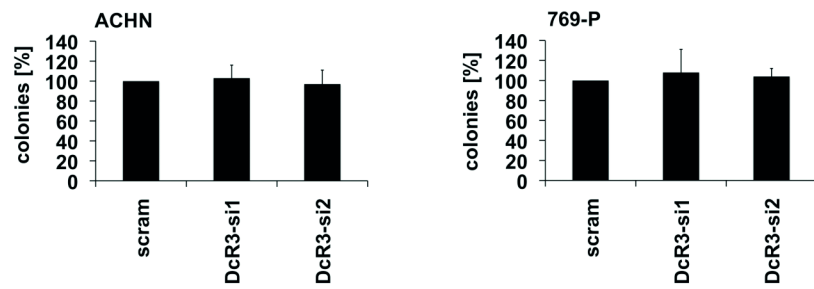

D

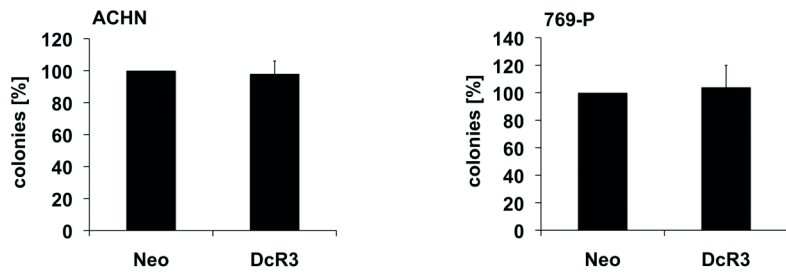

Supplement: Additional file 2: Figure S2 — DcR3 expression does not affect proliferation or clonogenicity in RCC. (A) Proliferation assay of ACHN and 769-P cells 24, 48 and 72 h after transfection with two different DcR3-specific siRNAs or a non-specific siRNA (scram). Cells were trypsinized and counted (mean ± SEM; n=3). (B) Proliferation assay of ACHN and 769-P cells stably overexpressing DcR3 or an empty vector control (neo) after 24, 48 and 72 h. Cells were trypsinized and counted (mean ± SEM; n=3). (C) Clonogenicity assay of ACHN and 769-P cells transfected with two different DcR3-specific siRNAs or a non-specific siRNA (scram). Cells were grown for 7–9 days, subsequently stained with crystal violet and colonies were counted (mean ± SEM; n=3). (D) Clonogenicity assay of ACHN and 769-P cells stably overexpressing DcR3 or an empty vector control (neo). Cells were grown for 7–9 days, subsequently stained with crystal violet and colonies were counted (mean ± SEM; n=3). [file 1476-4598-12-120-S2.pdf]

# Supplementary Figure 3

A

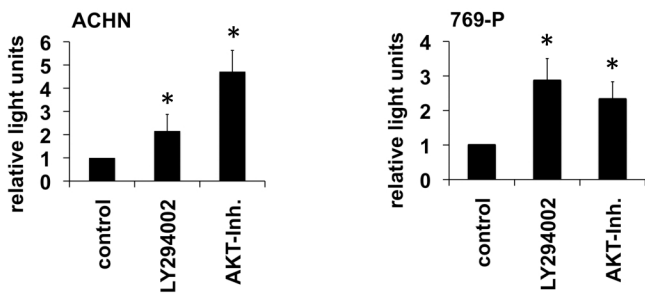

B

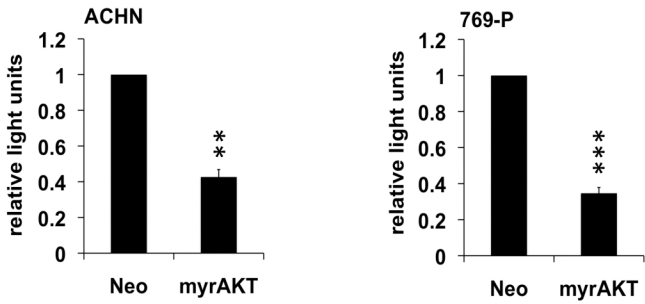

C

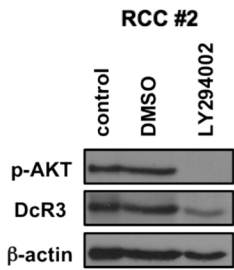

Supplement: Additional file 3: Figure S3 — NFAT mediated DcR3 expression is PI3K/AKT dependent. (A) Relative Forkhead response element–luciferase reporter activity of ACHN and 769-P cells 24 h after treatment with LY294002 (50 μM) or AKT-inhibitor IV (10 μM) (mean ± SEM; n=3; *p<0.05; T-test). (B) Relative Forkhead response element–luciferase reporter activity of ACHN and 769-P cells 24 h post transfection with myrAKT or an empty vector control (neo) (mean ± SEM; n=3; **p<0.01, ***p<0.001; T-test). (C) Representative immunoblot analysis of whole-cell lysates of ex vivo tissue 48 h after treatment with LY294002 (100 μM). [file 1476-4598-12-120-S3.pdf]
